# Treatment with 4-Methylpyrazole Modulated Stellate Cells and Natural Killer Cells and Ameliorated Liver Fibrosis in Mice

**DOI:** 10.1371/journal.pone.0127946

**Published:** 2015-05-29

**Authors:** Hyon-Seung Yi, Hyuk Soo Eun, Young-Sun Lee, Ju Yeon Jung, Seol-Hee Park, Keun-Gyu Park, Hueng-Sik Choi, Jae Myoung Suh, Won-Il Jeong

**Affiliations:** 1 Lab of Liver Research, Graduate School of Medical Science and Engineering, KAIST, Daejeon, 305–701, Republic of Korea; 2 Department of Internal Medicine, Korea University College of Medicine, Seoul, Republic of Korea; 3 Department of Internal Medicine, College of Veterinary Medicine, Seoul National University, Seoul, Republic of Korea; 4 Department of Internal Medicine, School of Medicine, Kyungpook National University, Daegu, Republic of Korea; 5 National Creative Research Initiatives Center for Nuclear Receptor Signals and Hormone Research Center, School of Biology Sciences and Technology, Chonnam National University, Gwangju, Republic of Korea; Kanazawa Medical University, JAPAN

## Abstract

**Background & Aims:**

Accumulating evidence suggests that retinol and its metabolites are closely associated with liver fibrogenesis. Recently, we demonstrated that genetic ablation of alcohol dehydrogenase 3 (ADH3), a retinol metabolizing gene that is expressed in hepatic stellate cells (HSCs) and natural killer (NK) cells, attenuated liver fibrosis in mice. In the current study, we investigated whether pharmacological ablation of ADH3 has therapeutic effects on experimentally induced liver fibrosis in mice.

**Methods:**

Liver fibrosis was induced by intraperitoneal injections of carbon tetrachloride (CCl_4_) or bile duct ligation (BDL) for two weeks. To inhibit ADH3-mediated retinol metabolism, 10 μg 4-methylpyrazole (4-MP)/g of body weight was administered to mice treated with CCl_4_ or subjected to BDL. The mice were sacrificed at week 2 to evaluate the regression of liver fibrosis. Liver sections were stained for collagen and α-smooth muscle actin (α-SMA). In addition, HSCs and NK cells were isolated from control and treated mice livers for molecular and immunological studies.

**Results:**

Treatment with 4-MP attenuated CCl_4_- and BDL-induced liver fibrosis in mice, without any adverse effects. HSCs from 4-MP treated mice depicted decreased levels of retinoic acids and increased retinol content than HSCs from control mice. In addition, the expression of α-SMA, transforming growth factor-β1 (TGF-β1), and type I collagen α1 was significantly reduced in the HSCs of 4-MP treated mice compared to the HSCs from control mice. Furthermore, inhibition of retinol metabolism by 4-MP increased interferon-γ production in NK cells, resulting in increased apoptosis of activated HSCs.

**Conclusions:**

Based on our data, we conclude that inhibition of retinol metabolism by 4-MP ameliorates liver fibrosis in mice through activation of NK cells and suppression of HSCs. Therefore, retinol and its metabolizing enzyme, ADH3, might be potential targets for therapeutic intervention of liver fibrosis.

## Introduction

Liver fibrosis is a response to wound healing process triggered by various types of chronic liver injuries. During this process, hepatic stellate cells (HSCs) produce major portions of extracellular matrix proteins including collagens in the liver [1]. Upon activation by fibrogenic stimuli *in vivo*, or prolonged cell culture *in vitro*, HSCs begin to lose their retinol lipid droplets and transform into myofibroblast like cells with simultaneous expression of α-smooth muscle actin (α-SMA) and production of collagens [1,2]. Many factors, including toxins, viruses, oxidative stress, necrosis, apoptosis, and growth factors, are involved in the activation of quiescent HSCs into myofibroblast like cells [3–6]. The activated HSCs migrate and accumulate at injured sites in the liver and synthesize excess amounts of extracellular matrix components, thereby leading to scarring events, the pathological hallmark of liver fibrosis [7].

HSCs also interact with various immune cells during liver fibrogenesis [8]. Several recent studies have demonstrated that HSCs can metabolize intracellular retinol deposits into retinoic acids via alcohol dehydrogenases (ADHs) and retinaldehyde dehydrogenases (RALDHs) which in turn modulate a diverse range of hepatic immune cells involved in liver fibrosis [9–13]. For example, the conversion of retinols to retinoic acids induces the expression of retinoic acid early inducible gene 1 (RAE1) in HSCs, a specific ligand for the natural killer group 2D (NKG2D) receptor of natural killer (NK) cells. This leads to the amelioration of liver fibrosis by enhancing the rate at which NK cells kill activated HSCs via increased expression of interferon-γ (IFN-γ) and tumor necrosis factor-related apoptosis inducing ligand (TRAIL) [11]. Retinoic acids in HSCs also stimulate interleukin-10 (IL-10) production by infused Gr1^+^CD11b^+^ bone marrow cells, subsequently suppressing HSC activation and liver fibrosis [13]. Yet in another example, HSCs promote the differentiation of naïve T cells into regulatory T cells in a retinoic acid-dependent manner [9]. Interestingly, our previous study demonstrated that ADH3 (ADH family class III) is a specific enzyme for retinol metabolism that functions not only in HSCs but also in NK cells and that genetic ablation of ADH3 decreased liver fibrosis by suppressing HSC activation while increasing NK cytotoxicity in carbon tetrachloride (CCl_4_)- and bile duct ligation (BDL)-induced models of fibrosis [14]. These results suggest that retinoic acids and ADH3 are important mediators of NK cell activity and liver fibrosis.

A pyrazole derivative, 4-methylpyrazole (4-MP), has been used medically as an antidote for methanol and ethylene glycol poisoning in the clinic owing to its inhibitory effects on the oxidation of ethanol and other alcohol analogues via suppression of ADHs [15–18]. In addition, 4-MP treatment can induce cytochrome P450 2E1 (CYP2E1) in rat liver, which can, in turn, affect xenobiotic responses and drug metabolism [19]. Interestingly, a recent investigation suggested that ADH-mediated retinol metabolism is necessary for rat liver regeneration, as 4-MP-mediated inhibition of cytosolic ADH activity led to the progressive inhibition of liver regeneration by suppressing hepatic retinol metabolism [20]. However, it was not clear whether treatment with 4-MP inhibits retinol metabolism in liver fibrosis or affects the activities of HSCs and NK cells by suppressing ADHs. In the present study, we investigated the effects of 4-MP on liver retinol metabolism and assessed its potential therapeutic effects to inhibit retinol metabolism and subsequent hepatic fibrogenesis in experimental models of liver fibrosis.

## Materials and Methods

### Animals

Male C57BL/6J mice were purchased from Jackson Laboratory (Bar Harbor, ME). Animals were maintained in a pathogen-free animal facility at the Korea Advanced Institute of Science and Technology. All of the animals received humane care according to the criteria outlined in the Guide for the Care and Use of Laboratory Animals published by National Institutes of Health. The animal protocol was approved by the Institutional Animal Care and Use Committee of the Korea Advanced Institute of Science and Technology (Permit Number: KA2009-13).

### Materials

CCl_4_, OptiPrep, 4-MP and type I Collagenase were obtained from Sigma-Aldrich (St. Louis, MO). Percoll and DNase I were purchased from GE Healthcare (Buckinghamshire, United Kingdom) and Roche (Indianapolis, IN), respectively.

### Induction of Liver Fibrosis in Mice

**Fig 1:**
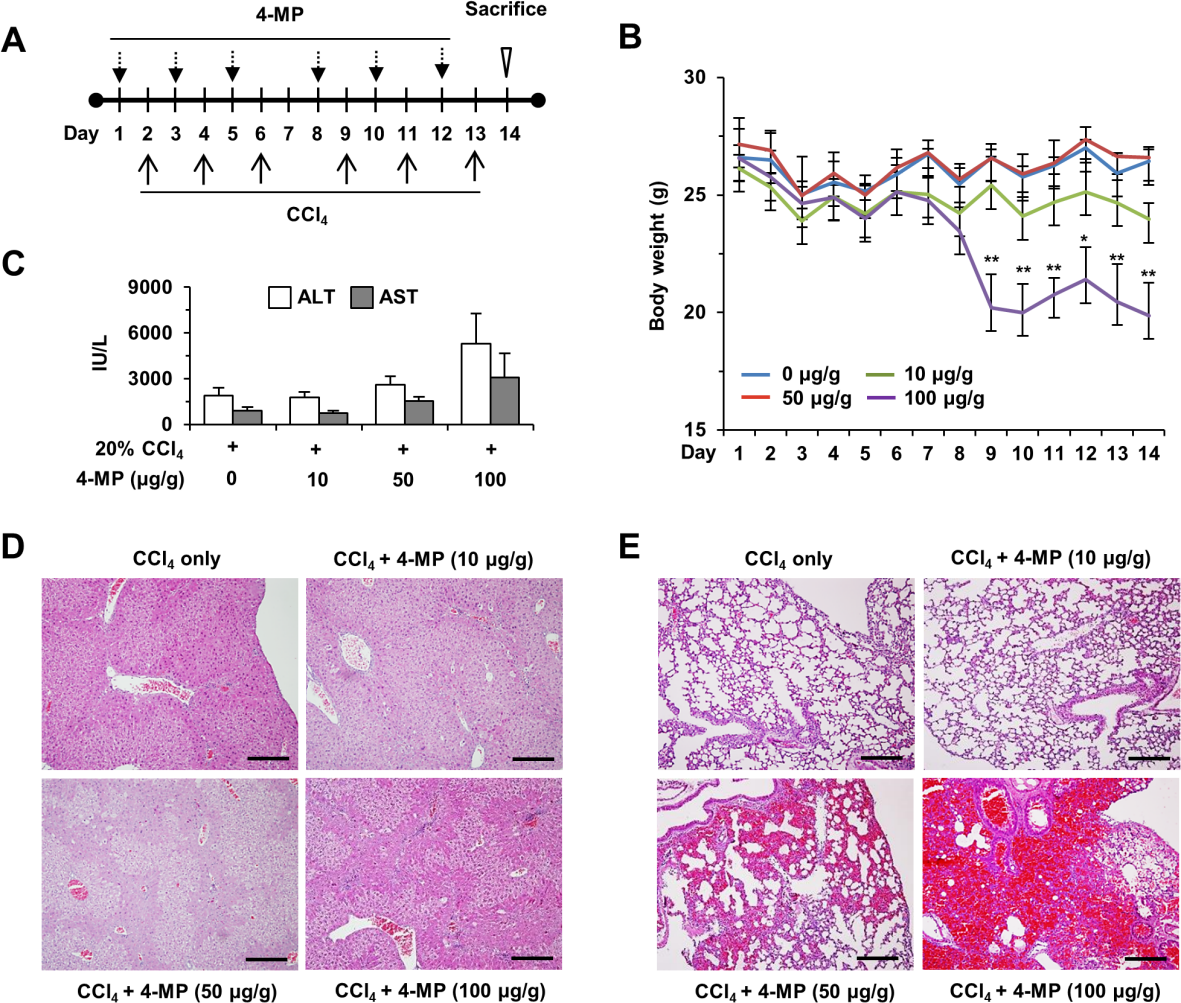
Treatment with 4-MP during CCl_4_-induced liver fibrosis in mice. Liver fibrosis was induced by intraperitoneal injection of CCl_**4**_ and different doses of 4-MP (10 to 100 μg/g) was administered for 2 weeks. (A) Experimental protocol of the injections with CCl_**4**_ and 4-MP. (B) Alteration of animal body weight during the study. (C) Serum levels of ALT and AST were measured. (D, E) Hematoxylin and eosin staining of liver and lung sections after administration of CCl_**4**_ and 4-MP (scale bar = 500 μm). The data are expressed as mean ± SEM. *P < 0.05, **P < 0.01 compared with the respective controls.

For the induction of CCl_4_-mediated liver fibrosis, age-matched (8-week-old) male C57BL/6J mice received intraperitoneal injections of CCl_4_ (20% CCl_4_ olive oil solution, 2 ml/kg of animal body weight) three times per week for 2 weeks (Fig 1A). Mice in the control group received an equal volume of olive oil (vehicle). Mice were sacrificed at 12 hours after the final injection. To generate cholestasis-induced liver fibrosis, age-matched (8-week-old) male C57BL/6J mice were underwent BDL [21]. Briefly, mice were anesthetized with isoflurane and BDL was performed after midline laparotomy. The common bile duct was ligated with 5–0 surgical silk and transected between the 2 distal ligations. Sham-operated mice had their common bile duct exposed and manipulated but not ligated or transected. For inhibition of ADH, 10 μg of 4-MP/g of animal body weight was injected intraperitoneally three times per week for 2 weeks (Fig 1A). For the NK cell activity assay, 2.5 μg polyinosinic-polycytidylic acid (poly I:C)/g of animal body weight (Sigma Aldrich, St. Louis, MO) was injected 12 hours after a single 4-MP challenge [22].

### Serum Biochemical Measurements

Serum was collected and assayed for alanine aminotransferase (ALT) and aspartate transaminase (AST) using kits purchased from IDEXX Laboratories (ME, USA). Serum levels of tumor necrosis factor-α (TNF-α), interleukin 6 (IL-6), monocyte chemotactic protein 1 (MCP-1) and IFN-γ were measured using an ELISA kit (Biosource International Inc, Camarillo, CA).

### Isolation of liver mononuclear cells (MNCs) and NK cells

Liver MNCs were isolated as described previously [12,23]. Briefly, liver tissues were dissociated by mashing through a 70 μm nylon mesh cell strainer (BD Falcon, Millville, NJ). The liver cell suspension was suspended in phosphate-buffered saline (PBS) and centrifuged at 42 g for 5 minutes to eliminate hepatocytes. The supernatant was collected, washed in PBS, and resuspended in 40% Percoll gradient (GE Healthcare, Buckinghamshire, United Kingdom) in PBS. The cell suspension was centrifuged at 1,200 g for 30 minutes at 4°C. The supernatant was removed by mechanical suction, and MNCs were collected, washed in PBS, and resuspended in RPMI-1640 medium. For the isolation of hepatic NK cells, CD3^-^ liver MNCs were separated from total liver MNCs by negative magnetic cell sorting (Miltenyi Biotec, Auburn, CA). NK1.1^+^CD3^-^ cells were then purified with anti-NK1.1 monoclonal antibodies by positive magnetic cell sorting. Approximately 95% of cells purified this way were NK1.1^+^CD3^-^ cells.

### Measurement of retinoids by HPLC

Cells were quantified and cellular extracts were prepared as described [12,23]. HPLC measurements were performed using a Hewlett–Packard 1100 HPLC equipped with a Zorbax Eclipse 5 μm XDB-C18 analytical column (250 X 4.6 mm; Agilent Technologies Inc, Palo Alto, CA). A linear gradient solvent system was used: 5% acetic acid aqueous solution/MeOH from 55:45 to 35:65 in 40 min; the flow rate was 1 mL/min. Peaks were detected by UV absorption (330 nm for retinol and 360 nm for the others) with a diode array detector. Acitretin was used as an internal standard. All standard reagents and solvents were purchased from Sigma-Aldrich (St. Louis, MO). LC-MS measurements were performed to double-check compounds on a Micromass/Waters LCT Premier Electrospray Time of Flight mass spectrometer coupled with a Waters HPLC system.

### Fluorescence-activated cell sorting (FACS) analyses

Isolated liver MNCs were stained with anti-CD45, anti-NK1.1, anti-CD3e, anti-TRAIL (BD Pharmingen, San Jose, CA), and anti-NKG2D antibodies (eBioscience, San Diego, CA). Non-specific antibody reactions were blocked by pre-incubating cells with anti-mouse CD16/32 mouse Fc blocker (BD Pharmingen, San Jose, CA). Stained cells were analysed using a BD LSR II Flow Cytometer (BD Pharmingen, San Jose, CA) and FlowJo software (Tree Star, Ashland, OR). A PE-conjugated anti-IFN-γ antibody (BD Pharmingen, San Jose, CA) was used for intracellular staining. Intracellular cytokine staining was performed after re-stimulation of the cells with phorbol-myristate acetate/ionomycin/brefeldin A for 5 hours using BD Cytofix/Cytoperm (BD Pharmingen, San Jose, CA).

### Staining

The liver and lung sections were stained with hematoxylin and eosin for histopathological evaluation and with 0.1% Sirius Red (Sigma-Aldrich, St. Louis, MO) to study collagen deposition. Collagen fiber deposition was quantified by imaging six randomly selected fields from each section with an Olympus BX51 microscope equipped with a CCD camera (Tokyo, Japan) followed by computer-assisted image analysis with DP2-BSW (Tokyo, Japan). Immunohistochemistry was performed with an anti-α-SMA) antibody (#M0851, Dako, Glostrup, Denmark). Antigen-antibody complexes were visualized with avidin-biotin peroxidase complex solution from an ABC kit (Vector Laboratories, Burlingame, CA) and DAB (Invitrogen, Eugene, OR). To identify apoptotic HSCs, double staining was performed with the anti-α-SMA antibody (Dako, Glostrup, Denmark) and an apoptosis detection kit (Roche, Indianapolis, IN). Immunofluorescence staining was performed with anti-α-SMA antibody (#M0851, Dako, Glostrup, Denmark), and then visualized with an Alexa Fluor 594-conjugated anti-mouse IgG secondary antibody (Invitrogen, Eugene, OR). The stained sections were mounted with aqueous based medium containing DAPI (Vector Laboratories, Burlingame, CA). All of the visual inspection was done by Olympus BX51 microscope (Olympus, Tokyo, Japan) equipped with a CCD camera and computer-assisted image analysis with DP2-BSW (Olympus, Tokyo, Japan).

### Western blotting

Liver tissues or isolated cells were homogenized in RIPA buffer (30 mmol/L Tris, pH 7.5, containing 150 mmol/L sodium chloride, 1 mmol/L phenylmethylsulphonyl fluoride, 1 mmol/L sodium orthovanadate, 1% Nonidet P-40, 10% glycerol, phosphatase and protease inhibitors) for protein extraction. Western blotting was performed with 30–50 μg protein using antibodies for β-actin (#A1978), α-SMA (#A2547) (Sigma-Aldrich, St. Louis, MO), TGF-ß1 (#3709S), phospho-pSMAD3 (#9520) (Cell Signaling, Danvers, MA), Class III ADH (#AB59134, Abcam, Cambridge, UK), ADH1 (#SC-22750, SantaCruz, Dallas, TX), and cytochrome P4502E1 (CYP2E1) (#AB1252, Merck Millipore, Darmstadt, Germany). The nitrocellulose membranes were treated with respective anti-rabbit or anti-mouse secondary antibody and the immuno-reactive protein bands were visualized using ECL detection system with a PhosphorImager (Thermo Scientific, Rockford, IL).

### Reverse transcription-polymerase chain reaction (RT-PCR) and quantitative real-time PCR

PCR analyses were performed and all primers used in this study are listed in Table 1. Total RNA was extracted from liver cells or tissues using TRIzol Reagent (Invitrogen, Eugene, OR) in accordance with the manufacturer’s instructions. The same quantity of total RNA was reverse-transcribed into complementary DNA (cDNA) using amfi-revert II cDNA synthesis Master Mix (Gendepot, Barker, TX). Quantitative real-time PCR was performed using SYBR Green Real-time PCR Master Mix (Toyobo, Osaka, Japan). The comparative Ct method was used to quantify transcripts normalized in respect to β-actin expression levels. Results were analysed by the ΔΔCt method [14]. Values are expressed as fold change relative to the control.

**Table 1:** Primers used in RT-PCR and quantitative real-time PCR (mouse).

Genes	Forward (5’-3’)	Reverse (5’-3’)	PCR product (base pairs)
α-SMA	CTGACAGAGGCACCACTGAA	GAAGGAATAGCCACGCTCAG	287
COL1A1	TCCTCCAGGGATCCAACGA	GGCAGGCGGGAGGTCTT	148
TGF-β1	TTGCTTCAGCTCCACAGAGA	TGGTTGTAGAGGGCAAGGAC	182
IL-6	TCCATCCAGTTGCCTTCTTG	TTCCACGATTTCCCAGAGAAC	166
MCP-1	TCAGCCAGATGCAGTTAACGC	TCTGGACCCATTCCTTCTTGG	184
NKG2D	GCATTGATTCGTGATCGAAA	GCCACAGTAGCCCTCTCTTG	374
IFN-γ	AGACATCTCCTCCCATCAGCAG	TAGCCAAGACTGTGATTGCGG	158
TRAIL	CCCTGCTTGCAGGTTAAGAG	GGCCTAAGGTCTTTCCATCC	219
Perforin	GATGTGAACCCTAGGCCAGA	GGTTTTTGTACCAGGCGAGA	162
β-actin	AGAGGGAAATCGTGCGTGAC	CAATAGTGATGACCTGGCCGT	148

### Statistical Analysis

Data are presented as means ± SEM. To compare values from two or more groups, Student’s *t* test or one-way analysis of variance was performed. A value of P < 0.05 was considered as statistically significant.

## Results

### 4-MP Toxicity assay in CCl_4_-induced liver injury and NK cell activity

To determine whether 4-MP has a toxic effect on CCl_4_-induced liver injury or affects NK cell activity, CCl_4_ was injected into mice, together with various doses of 4-MP, for 2 weeks (Fig 1A). Throughout the experiment, treatments with CCl_4_ successfully induced liver fibrosis without mortality, but mice treated with 50 or 100 μg 4-MP/g of animal body weight showed significant weight loss, hepatotoxicity, or pulmonary hemorrhage (Fig 1B–1E). However, mice co-treated with 10 μg 4-MP/g of animal body weight did not show any toxic reaction during liver fibrogenesis.

**Fig 2:**
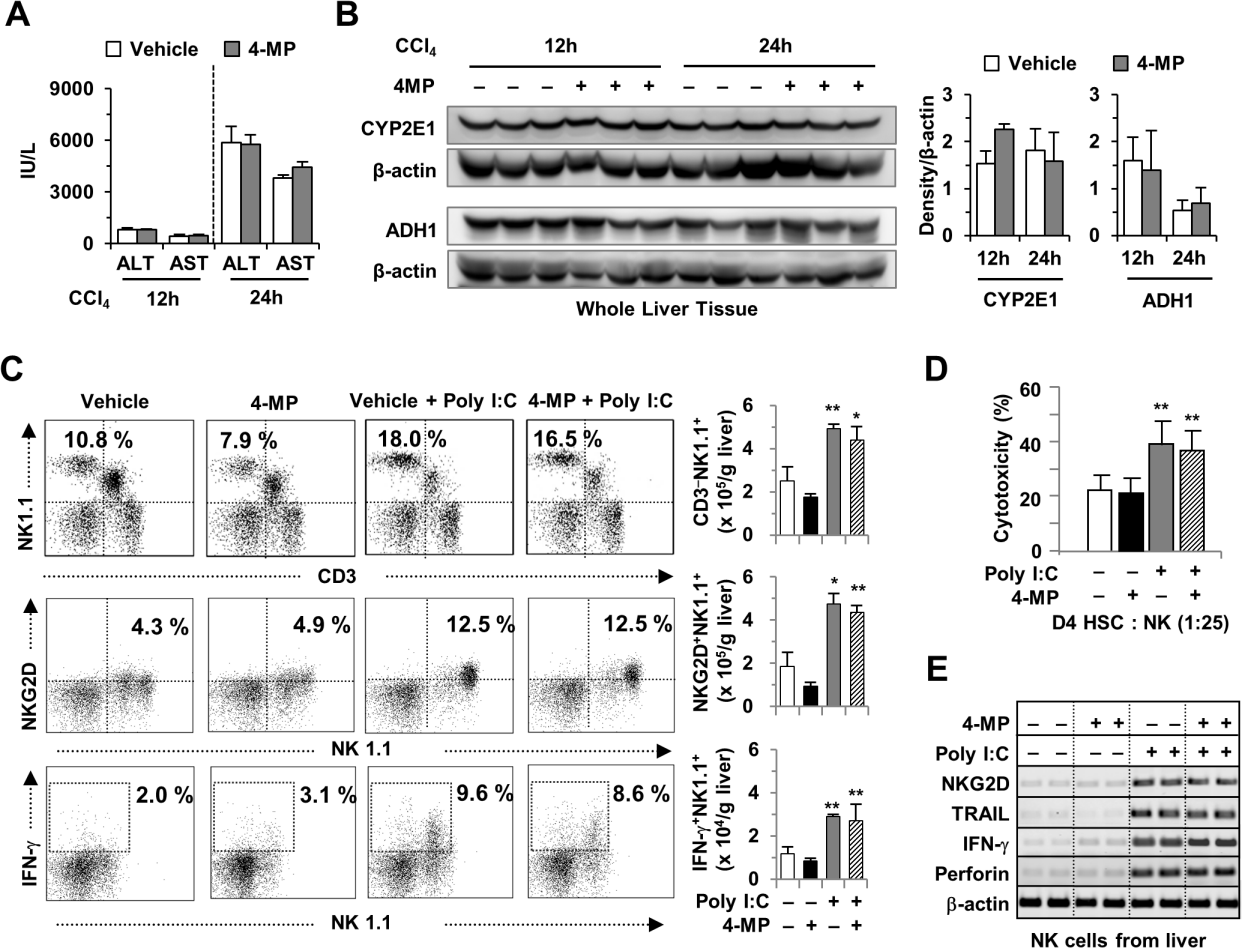
Treatment with 4-MP did not alter CCl_4_-induced liver injury or NK cell activity in the liver. A single dose of CCl_**4**_ (20% CCl_**4**_ in olive oil, 2 ml/kg body weight) was administered to wild-type mice at 12 or 24 h after the treatment with 10 μg 4-MP/g of body weight. (A) Serum levels of ALT and AST. (B) Western blotting for CYP2E1 and ADH1 and the quantified data. (C) FACS analyses were performed on liver mononuclear cells from poly I:C and/or 4-MP-treated mice using antibodies against NK1.1, CD3, CD45, NKG2D and IFN-γ. (D) Cytotoxicity assays on 4 days old HSCs (D4 HSC). (E) Gene expression analyses on freshly isolated liver NK cells. The data are expressed as mean ± SEM. *P < 0.05, **P < 0.01 compared with the respective controls.

Next, we tested whether a dose of 10 μg 4-MP/kg of animal body weight has toxic effects in conjunction with a single treatment of CCl_4_. As shown in Fig 2A, treatment with 4-MP did not induce significant changes in serum levels of ALT or AST after a single CCl_4_ challenge. Moreover, Western blotting demonstrated that 4-MP did not have any effect on the expression of CYP2E1 and ADH1 in the liver of CCl_4_-challenged mice (Fig 2B). We next examined the effects of 4-MP on poly I:C-mediated activation of NK cells, as previously reported [24,25]. By FACS analyses, treatment with 4-MP did not induce significant differences in the frequencies or numbers of liver NK cells (CD3^-^NK1.1^+^, NKG2D^+^NK1.1^+^ or IFN-γ^+^NK1.1^+^), NK cell cytotoxicity against activated 4-day cultured HSCs (D4 HSCs), or gene expression in NK cells, compared to NK cells from non-4-MP-treated mice (Fig 2C–2E). Based on these data, we concluded that treatment with 10 μg 4-MP/g of animal body weight had no adverse effects on CCl_4_-induced liver injury or on the activity of NK cells.

### 4-MP-mediated ADH inhibition ameliorates CCl_4_-induced liver fibrosis in mice

**Fig 3:**
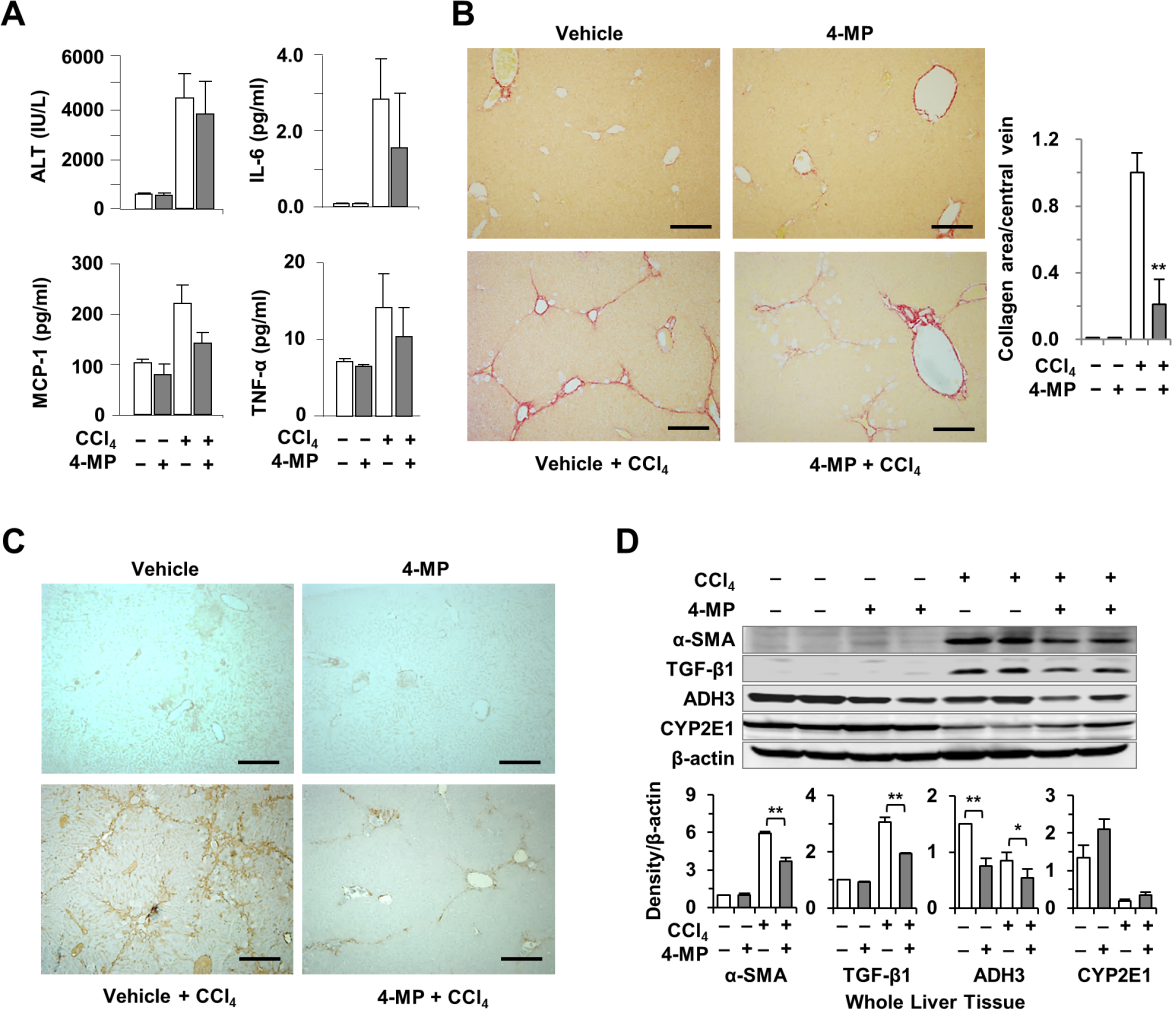
Treatment with 4-MP attenuated CCl_4_-induced liver fibrosis in mice. Fibrosis was induced with serial administrations of CCl_**4**_ and the animals were concurrently treated with 4-MP for 2 weeks. (A) Serum levels of ALT, IL-6, MCP-1, and TNF-α. (B) Sirius red staining for collagen (scale bar = 500 μm). (C) Immunohistochemical staining for α-SMA demonstrating activated HSCs (scale bar = 500 μm). (D) Western blotting for α-SMA, TGF-β1, ADH3, and CYP2E1 and the respective quantification of proteins. The data are expressed as mean ± SEM. *P < 0.05, **P < 0.01 compared with the respective controls.

Based on the above data, we tested whether treatment with 4-MP has beneficial effects on liver fibrosis in mice. To investigate the anti-fibrotic effects of 4-MP, mice were co-injected with CCl_4_ and 4-MP for 2 weeks. As shown in Fig 3A, treatment with 4-MP did not cause significant alterations in serum levels of ALT, IL-6, MCP-1 and TNF-α after CCl_4_ challenge for 2 weeks. However, treatment with 4-MP significantly reduced the accumulation of collagen fibers (Fig 3B) and the expression of α-SMA (Fig 3C). Moreover, Western blotting revealed that the protein levels of α-SMA, transforming growth factor (TGF)-β1, and ADH3 in the liver of 4-MP plus CCl_4_-injected mice were significantly lower than those of CCl_4_-treated mice (Fig 3D). These data indicated that treatment with 4-MP inhibited the progression of liver fibrosis in mice via suppression of ADH3.

### Treatment with 4-MP inhibits activation and retinol metabolism of HSCs in CCl_4_-induced liver fibrosis

**Fig 4:**
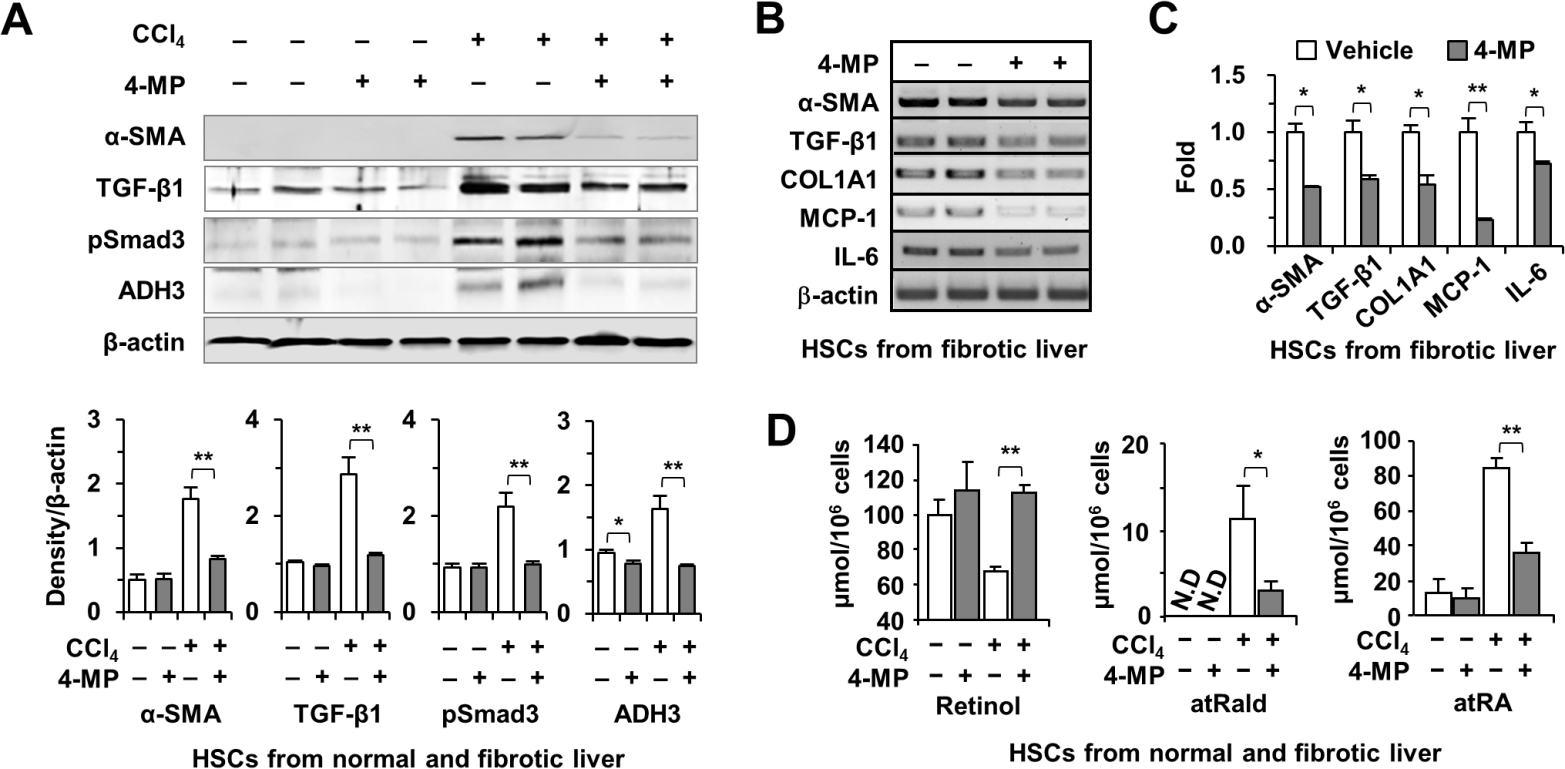
Inhibition of retinol metabolism by 4-MP suppresses HSC activation in CCl_4_-induced liver fibrosis. Mice were concurrently treated with CCl_**4**_ and 4-MP for 2 weeks and HSCs were isolated from the treated mice. (A) Representative Western blot images for α-SMA, TGF-β1, pSmad3, and ADH3 in isolated HSCs and quantification of respective images. Semi-quantitative PCR (B) and qPCR (C) for α-SMA, TGF-β1, COL1A1, MCP-1, and IL-6 in isolated HSCs. (D) Retinol and their metabolites, all trans-retinaldehyde (atRald) and retinoic acid (atRA), in isolated HSCs. Treatment with 4-MP increased retinol levels and reduced its metabolites significantly. The data are representative of three independent experiments and are expressed as mean ± SEM. *P < 0.05, **P < 0.01 compared with the respective controls.

To further investigate the effects of 4-MP in liver fibrosis, we isolated and examined primary HSCs from liver tissue. Interestingly, the protein levels of α-SMA, TGF-β1, pSmad3, and ADH3 were markedly down-regulated in HSCs isolated from 4-MP and CCl_4_-co-injected mice compared with HSCs isolated from CCl_4_-treated mice (Fig 4A). Moreover, RT-PCR and quantitative real-time PCR analyses revealed that HSCs from CCl_4_- and 4-MP-co-treated mice had lower expression of α-SMA, TGF-β1, type I collagen α1 (COL1A1), MCP-1 and IL-6 than HSCs from CCl_4_-treated mice (Fig 4B and 4C). Of note, a previous study of CCl_4_-treated rats reported increased levels of retinoic acid accompanied by decreased retinol contents in the liver [26]. Consistent with the previous report, liver injury by CCl_4_ decreased retinol levels but increased levels of their metabolites, including all trans-retinaldehyde (atRald) and all trans-retinoic acid (atRA) in HSCs isolated from CCl_4_-treated mice (Fig 4D). However, treatment with 4-MP inhibited retinol metabolism, leading to increased levels of retinols and decreased levels of their metabolites in HSCs isolated from CCl_4_-induced fibrotic livers compared with HSCs isolated from non-4-MP-treated mice (Fig 4D). In non-fibrotic livers, treatment with 4-MP did not alter the content of retinols or their metabolites in HSCs. Taken together, 4-MP-mediated inhibition of retinol metabolism leads to decreased HSC activation in CCl_4-_induced liver fibrosis.

### 4-MP treatment increases IFN-γ production in liver NK cells, resulting in increased HSC apoptosis

**Fig 5:**
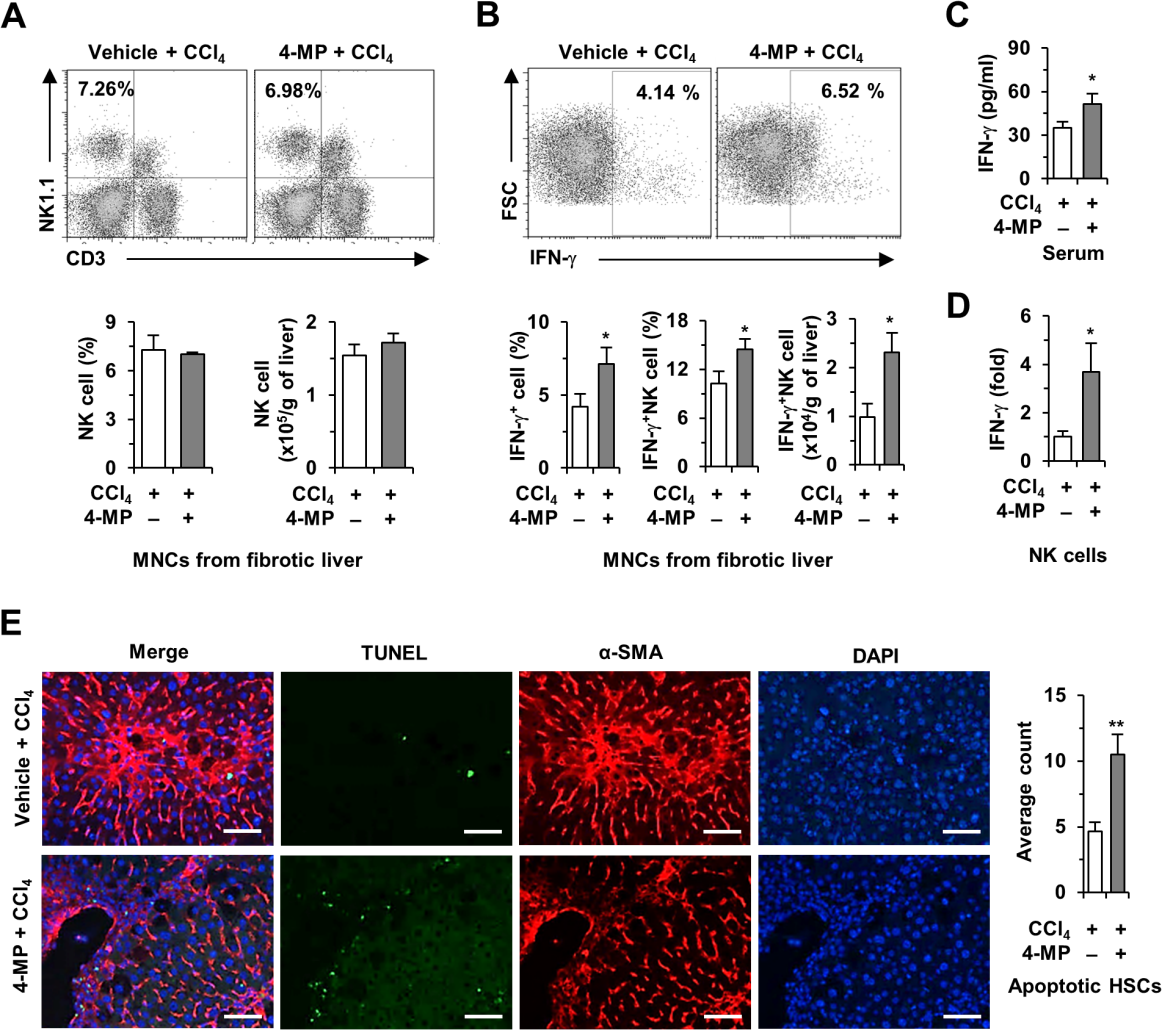
Treatment with 4-MP increased production of IFN-γ by NK cells in CCl_4_-treated mice. Hepatic fibrosis was induced with CCl_**4**_ and the mice were concurrently treated with 4-MP for 2 weeks. (A and B) FACS analyses of isolated mononuclear cells to assess the frequency of NK cells and IFN-γ production, respectively. (C) Serum levels of IFN-γ. (D) qPCR for IFN-γ in isolated NK cells. (E) Fluorescent TUNEL staining to demonstrate apoptosis in activated HSCs after treatment with 4-MP and the respective quantified data (scale bar = 25 μm). The data are representative of three independent experiments and are presented as mean ± SEM. *P < 0.05, **P < 0.01 when compared with the respective controls.

Next, we investigated the effects of 4-MP on liver NK cells in CCl_4_-induced liver fibrosis. By FACS analysis, there was no significant difference in the frequency of CD3^-^NK1.1^+^ cells (NK cells) between groups (Fig 5A), but the frequency and number of IFN-γ-producing NK cells were significantly increased in 4-MP-treated fibrotic mice compared with vehicle-treated fibrotic mice (Fig 5B). Consistent with the FACS analyses, serum levels of IFN-γ and its expression in NK cells in CCl_4_- and 4-MP-co-treated mice were significantly increased compared with CCl_4_-treated mice (Fig 5C and 5D). In addition, immunostaining revealed that apoptotic HSCs were significantly increased in the livers of CCl_4_ and 4-MP-co-treated mice (Fig 5E). In agreement with previous reports [14,23], these data suggest that the amelioration of CCl_4_-induced liver fibrosis by 4-MP may be, in part, due to enhanced cytotoxicity of IFN-γ-producing NK cells against activated HSCs.

### 4-MP treatment ameliorates BDL-induced liver fibrosis in mice

**Fig 6:**
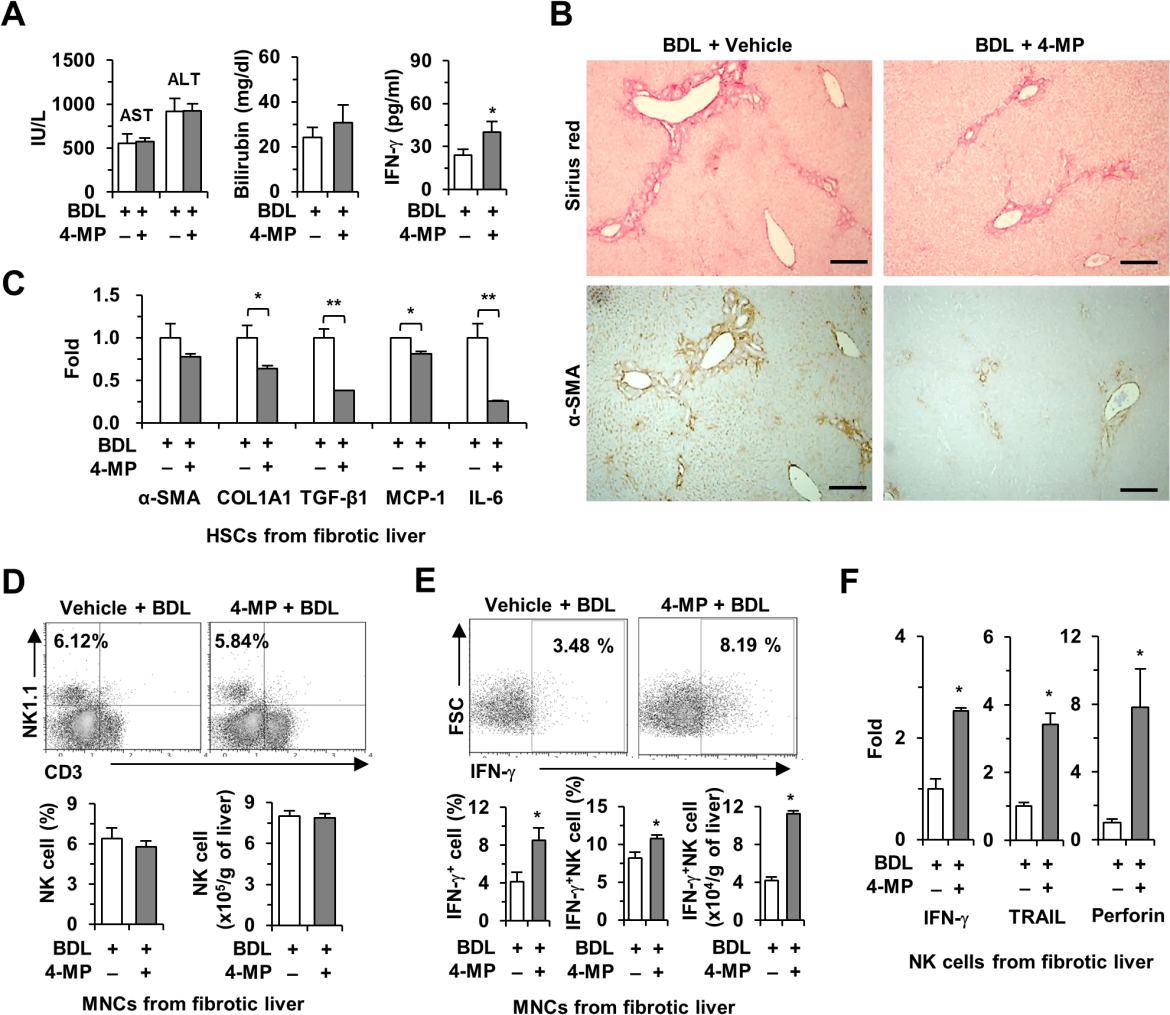
Treatment with 4-MP ameliorated BDL-induced liver fibrosis in mice. Liver fibrosis was induced by BDL and treated with 4-MP for 2 weeks. (A) Serum levels of AST, ALT, total bilirubin, and IFN-γ. (B) Liver sections were stained with Sirius red for collagen and α-SMA for activated HSCs after treatment with 4-MP (scale bar = 800 μm). (C) qPCR for α-SMA, COL1A1, TGF-β1, MCP-1 and IL-6 in isolated HSCs (D and E). Isolated liver mononuclear cells were subjected to FACS analyses to assess the frequency and IFN-γ production of NK cells. (F) qPCR for IFN-γ, TRAIL, and perforin in isolated NK cells from 4-MP treated BDL mice. The data are representative of three independent experiments and are presented as mean ± SEM. *P < 0.05, **P < 0.01 when compared with the respective controls.

To address whether the beneficial effects of 4-MP may be applicable to other models of liver fibrosis, we induced liver fibrosis by common BDL in mice. Throughout the 2-week-long BDL experiment, there was no case of mortality. As shown in Fig 6A, treatment with 4-MP did not cause significant changes in serum levels of AST, ALT or bilirubin 2 weeks after BDL, but it significantly increased serum levels of IFN-γ compared with non-4-MP-treated mice (Fig 6A). In liver tissues, liver fibrosis had developed in BDL-operated mice, whereas the accumulation of collagen fibers and the expression of α-SMA were markedly reduced in BDL-operated mice co-treated with 4-MP compared with mice subjected to BDL without 4-MP treatment (Fig 6B). In quantitative real-time PCR analyses, the expression of α-SMA, COL1A1, TGF-β1, MCP-1 and IL-6 was also suppressed in freshly isolated HSCs from BDL-operated mice co-treated with 4-MP compared with BDL-operated mice (Fig 6C). FACS analyses further showed that while there was no difference in the frequency of liver NK cells between groups (Fig 6D), treatment with 4-MP increased the population of IFN-γ-producing NK cells compared with NK cells from BDL-operated mice (Fig 6E). Furthermore, the expression of IFN-γ, TRAIL, and perforin was significantly increased in freshly isolated NK cells from BDL-operated mice co-treated with 4-MP (Fig 6F). Similar to our results from the CCl_4_-induced liver fibrosis model, these data demonstrate that 4-MP-mediated inhibition of retinol metabolism also has anti-fibrotic effects on BDL-induced liver fibrosis by suppressing HSC activation and enhancing NK cell activity in mice.

## Discussion

A number of studies have demonstrated that retinol and its metabolites are strongly linked to a variety of liver diseases including hepatic fibrosis [12,14,27–29]. However, the role of retinol and its metabolites in HSCs and liver fibrosis is not fully understood. Recent studies have shown that retinol treatment inhibits DNA synthesis in proliferating HSCs *in vitro* and ameliorates liver fibrosis in rats [30,31]. In apparent contrast, Kluwe et al. reported that the absence of HSC lipid droplets in lecithin retinol acyltransferase (LRAT)-deficient mice did not increase liver fibrosis, indicating that retinol storage itself was not associated with liver fibrosis [27]. Recently, we demonstrated that ADH3 is the major retinol-metabolizing enzyme in non-parenchymal cells, whereas hepatocytes express various types of retinol-metabolizing enzymes, including ADH1 and ADH3 [14]. Our study showed that genetic ablation of ADH3 in HSCs and NK cells attenuated liver fibrosis by inhibiting HSC activation and increasing IFN-γ production by NK cells [14]. In the present study, we demonstrated that pharmacological suppression of ADH3 by 4-MP also ameliorates CCl_4_- and BDL-induced liver fibrosis in mice by increasing IFN-γ production by NK cells and by suppressing HSC activation. Our studies depicted that both genetic and pharmacological ablation of ADH3 can attenuate liver fibrosis by regulating the functions of HSCs and NK cells.

Although 4-MP treatment has pleiotropic effects in the liver, such as CYP2E1 expression, it has been used as an antidote to methanol and ethylene glycol poisoning in human patients and to study alcohol metabolism in various experimental models [15–19]. In addition to the inhibitory role of 4-MP in alcohol metabolism, our previous study demonstrated that treatment with 4-MP could suppress retinol metabolism in HSCs and NK cells in an ADH3-dependent manner in vitro [14]. Another study showed that 4-MP treatment inhibited retinol metabolism in a dose-dependent manner, leading to decreased liver regeneration in a partial hepatectomy mouse model [20]. However, there have been no studies on the anti-fibrotic effects of 4-MP on liver fibrosis or on adequate dose of 4-MP for preventing liver fibrosis in mice. Here, for the first time, we tested various doses of 4-MP in the context of liver fibrogenesis in mice using previous reports as a reference [20,32,33]. As shown in Fig 1, chronic treatment with high doses of 4-MP (50 and 100 μg 4-MP/g of animal body weight for 2 weeks) induced several toxic effects in mice subjected to CCl_4_-induced liver fibrosis, including decreased body weight and increased injuries in the liver and lung. These adverse effects may be due to suppressed hepatocyte regeneration from the CCl_4_-induced liver injury and abnormal coagulopathy in the lung through inhibited retinol metabolism, respectively [20,34]. However, a ten-fold lower dose of 10 μg 4-MP/g of animal body weight did not show any toxic effects in mice treated for 2 weeks. Treatment with 10 μg 4-MP/g of animal body weight also did not affect CCl_4_-mediated liver injury or NK cell activity compared with vehicle-treated mice (Fig 2). Furthermore, treatment with 4-MP successfully attenuated CCl_4_- and BDL-induced liver fibrosis by suppressing HSC activation and enhancing NK cell cytotoxicity against activated HSCs (Figs 3–6). Consistent with our results, other studies have also demonstrated that treatment with 10 μg 4-MP/g of animal body was sufficient to inhibit methanol, ethanol and ethylene glycol metabolism via suppression of ADH enzyme activity in rodents and humans [32,33,35]. These data suggest that treatment with 10 μg 4-MP/g of animal body weight may represent a safe and efficacious therapeutic window for not only metabolic inhibition of alcohol analogues but also prevention of liver fibrosis.

Regardless of etiology, loss of retinol lipid droplets and expression of α-SMA are the hallmark signatures of HSC activation [1,2]. Previous studies reported that retinoic acids could activate the production of TGF-β1 in HSCs, subsequently leading to a more fibrotic condition of HSCs in an autocrine loop [28,36]. Consistently, our previous study demonstrated that levels of retinol metabolites, including retinoic acids, were increased in fibrotic livers and activated HSCs *in vitro*, whereas suppressed retinol metabolism by genetic ablation of ADH3 attenuated liver fibrosis and HSC activation [14]. In addition, 4-MP-mediated suppression of ADH3 expression might be due to decreased metabolism of retinols into retinoic acids in HSCs because direct retinoic acid treatment increased ADH3 expression in mouse HSCs and human hTERT cells [14]. Our data support the notion that pharmacological suppression of ADH3 reduced the production of retinoic acids in HSCs, resulting in attenuated expression of fibrotic mediators such as α-SMA, TGF-β1 and ADH3 in HSCs from CCl_4_- and BDL-induced fibrotic livers. These data suggest retinol metabolism in HSCs might be involved in the pathogenesis of liver fibrosis [11,12,26]. In contrast to the pro-fibrotic effects of retinoic acids, several studies have reported that retinol and its metabolites ameliorated liver fibrosis and HSC activation [29,37]. Therefore, the exact function of retinol and its metabolites should warrant further investigation.

Retinoids, including retinol and its metabolites, are considered important regulators of immune responses [38]. Retinoids also influence the population and cytotoxic activities of NK cells in spleen and peripheral blood cells [39]. However, recent evidence suggests that liver NK cells can kill or suppress activated HSCs in an IFN-γ-dependent manner, subsequently suppressing liver fibrosis [8,40,41]. Our previous study demonstrated that ADH3-mediated retinol metabolism resulted in the suppression of IFN-γ production by NK cells, thereby leading to the acceleration of liver fibrosis through increasing the survival rates of activated HSCs [14]. In addition, one study reported that retinoic acid treatment decreased IFN-γ secretion by CD4 T cells in mice [42]. These results suggest that retinoic acid negatively regulates IFN-γ production in immune cells. Similarly, in the present study, 4-MP-mediated inhibition of retinol metabolism enhanced IFN-γ production by NK cells isolated from mice with CCl_4_- or BDL-induced liver fibrosis (Figs 5B and 6E). These data were closely related to a higher rate of HSC apoptosis and lower levels of liver fibrosis (Fig 5E). In contrast, NK cells from vitamin A-deficient rats showed low cytotoxic activity against Yac-1 cells; the cytotoxicity of NK cells was effectively restored by retinoic acid supplementation [43]. These contradictory results suggest that NK cells may show different responses to retinoids in their cytotoxicity against target cells. One explanation may be that exogenous (external supplements) and endogenous (stored in HSCs) retinoids may have different effects on the population and activity of NK cells.

In the current study, we determined an optimum dose of 4-MP for treatment of liver fibrosis without significant adverse effects, and then confirmed the effect of this dosage using *in vivo* models of liver fibrosis. We also evaluated whether treatment with 4-MP has beneficial effects on the inhibition of retinol metabolism or anti-fibrotic potential using CCl_4_- and BDL-induced mouse models of liver fibrosis. We found that 4-MP-mediated inhibition of retinol metabolism suppressed HSC activation and increased NK cytotoxicity against activated HSCs, subsequently ameliorating liver fibrosis. Therefore, our data suggest that retinol and its metabolizing enzyme, ADH3, in HSCs are potential targets for therapeutic intervention of liver fibrosis.
